# Collagen Autoantibodies and Their Relationship to CCP Antibodies and Rheumatoid Factor in the Progression of Early Rheumatoid Arthritis

**DOI:** 10.3390/antib6020006

**Published:** 2017-04-05

**Authors:** Senga F. Whittingham, Alex Stockman, Merrill J. Rowley

**Affiliations:** 1 Department of Biochemistry and Molecular Biology, Monash University, Clayton VIC 3800, Australia; senga.whittingham@monash.edu; 2 Rheumatology Unit, Royal Melbourne Hospital, Grattan Street, Parkville VIC 3050, Australia; alex@stockman.net.au

**Keywords:** rheumatoid arthritis, collagen antibodies, anti-citrullinated protein antibodies, rheumatoid factor, immune complexes

## Abstract

Serum autoantibodies to cyclic citrullinated peptides (anti-CCP) and rheumatoid factor (RF) are important markers for diagnosis and prognosis of rheumatoid arthritis (RA), but their autoantigens are not cartilage-specific. Autoantibodies to joint-specific type II collagen (CII) also occur in RA, and monoclonal antibodies of similar specificity induce collagen antibody-induced arthritis in animals, but their role in RA is uncertain. We utilized an enzyme-linked immunosorbent assay (ELISA) with the CB10 peptide of CII to compare the frequency of autoantibodies with those of anti-CCP and RF in stored sera from a prospective study of 82 patients with early RA to examine the outcome, defined as remission (*n* = 23), persisting non-erosive arthritis (*n* = 27), or erosions (*n* = 32). Initial frequencies of anti-CB10, anti-CCP and RF were 76%, 54%, and 57% in RA, and 4%, 0%, and 9% in 136 controls. The frequency of anti-CB10 was unrelated to outcome, but anti-CCP and RF increased with increasing severity, and the number of autoantibodies mirrored the severity. We suggest RA is an immune complex-mediated arthritis in which the three antibodies interact, with anti-CII inducing localized cartilage damage and inflammation resulting in citrullination of joint proteins, neoepitope formation, and a strong anti-CCP response in genetically-susceptible subjects, all amplified and modified by RF.

## 1. Introduction

Rheumatoid arthritis is a chronic inflammatory arthritis of unknown cause. Dating back to the late 1940s when rheumatoid factor (RF) was first detected in patients with rheumatoid arthritis, circumstantial evidence suggested the perpetrator was autoimmune. However, precise early diagnosis is difficult, and clinical diagnosis is made by rheumatologists based on exclusion of other possible diagnoses and consideration of the fulfilment of criteria proposed by the American Rheumatism Association (ARA) in 1954 and revised in subsequent years [1,2,3,4]. These criteria have defined the disease more precisely and attracted widespread research on the clinical, histologic, immunologic, pharmacologic, and genetic aspects of RA, as well as addressing controversial issues regarding the understanding of the disease. The findings have largely substantiated the view that the mechanism underlying RA is autoimmune, but the identity of the joint-specific autoantigen involved in the failure of immunological tolerance is uncertain. 

RF as a diagnostic marker of RA may precede clinical symptoms by many years [5] and is associated with more severe disease and poor prognosis. Evidence of synthesis of RF within the joint, its presence in immune complexes with IgG, and low levels of complement in synovial fluid and cartilage suggest immune complexes may be an integral part of the crippling inflammation [6,7,8,9,10,11]. However, the association between RF and RA remains unclear. It is not specific for RA being found at low titre in chronic infection, in healthy subjects following immunization, and in primary Sjögren’s syndrome and other connective tissue diseases in the absence of arthritis [12,13,14]. Moreover, its antigenic determinant is the Fc region of immunoglobulin G (IgG), which is not a structural component of joints. 

Antibodies to citrullinated proteins (ACPA), routinely demonstrated as antibodies to cyclic citrullinated peptides (anti-CCP), have become established as major autoantibodies in RA and appear to be particularly associated with erosive arthritis [15]. Citrulline is derived by deimination of arginine by the enzyme peptidyl-arginine-deiminases (PADI) that are found in macrophages and neutrophils [16]. Tests for anti-CCP have been developed that can detect ACPA reactive with a range of citrullinated proteins [17]. The recognised protein antigens have rarely been identified, and vary in specificity and affinity between individuals, but ACPA reactive with citrullinated fibrinogen, alpha enolase, vimentin, and collagen type II (CII) have been the most frequently studied [18,19,20]. Like RF, ACPA precede the onset of clinical symptoms of RA [21,22,23], are produced within the joint [24,25], and are present at high titre when arthritis is severe [26]. When first reported, ACPA and anti-CCP appeared to have particular specificity for RA, but more recently the antibodies have been found in other diseases [27,28,29]. The ACPA response is, to a substantial degree, determined by the alleles of histocompatibility leucocyte antigen (HLA)-DRB1 that represent the RA-associated shared epitope (SE) [30]. The SE binds strongly to citrulline for T cell presentation [31] and ACPA production is presumed to be a response to neoepitopes resulting from citrullination. 

Although neither RF, nor most of the known anti-citrullinated protein antigens are confined to the joint, one potential joint-specific autoantigen is type II collagen, first proposed as an autoantigen in RA by Steffen in 1970 [32]. Antibodies to CII have been shown repeatedly in both serum and synovial fluid particularly early in the disease, although there may be a decline in serum levels of antibodies as the disease progresses [33,34,35]. Anti-CII is synthesized within the joint [36], and immune complexes containing collagen have been described in synovial fluid [9,37,38]. In animals, CII is arthritogenic and injection of native CII in adjuvant induces collagen-induced arthritis (CIA) characterized by antibodies to CII and an inflammatory polyarthritis [39,40]. Variability in expression of arthritis is linked to the expression of particular class II major histocompatibility (MHC) alleles [41] and depends on an intact immune system: animals that are B cell deficient [42] or deficient in complement [43] are protected. Moreover, monoclonal antibodies (mAb) to CII derived from mice with CIA can induce collagen antibody-induced arthritis (CAIA) in naïve mice. CAIA is characterised by inflammation, formation of pannus, and erosions of bone not unlike that observed in RA [44]. This model disease does not require T cell help, and is an informative model of how antibodies lead to arthritis. The arthritis is not MHC-restricted, can be induced in most strains of mice, and represents a model of the effector arm of CIA, but depends on the specificity of the antibodies used. These arthritogenic mAb recognize epitopes on CII that share a common amino acid motif, a triplet of arginine-glycine-hydrophobic acids, and map to surface-exposed regions on the collagen fibrils that are accessible for antibody binding [44]. The epitopes are conserved, and are also recognised by antibodies from rats [45,46,47,48] and from humans with RA [36,46]. Interestingly, the major arthritogenic epitopes on the collagen fibrils contain surface-exposed arginines that can also become citrullinated [49] and mAb reactive with these citrullinated epitopes may be arthritogenic themselves, or induce more severe arthritis when injected with subclinical doses of anti-CII [50].

Taken together, these studies suggest that all three autoantibody populations could play a role in the development and progression of RA. However, although there have been several studies of the utility of measurement of serum RF or anti-CCP to study the outcome of early RA, none of these studies has included anti-CII, as there is no routine diagnostic test for anti-CII currently available. We have previously used an ELISA in which the intact CII molecule was replaced with the triple helical CB10 fragment of the native CII molecule to detect antibodies to CII [51]. CB10 is one of the two major peptides derived by digestion of CII with cyanogen bromide that contain important arthritogenic epitopes in animal studies, and represents 30% of intact CII. Using this CB10 ELISA, we detected autoantibodies in 84 (88%) of 96 patients with RA compared with 24% using intact CII. In contrast, anti-CB10 was present in only 4 of 33 (12%) patients with psoriatic arthritis, 2 of 34 (10%) with osteoarthritis, and three of 93 (3%) blood donors [51]. CB11, the second peptide that contains major arthritogenic epitopes in animal studies was also highly reactive, but the disease specificity of the response was reduced. 

In that study, the patients had established RA, and were attending the Rheumatology Clinic of the Royal Melbourne Hospital, a tertiary referral centre. All patients fulfilled the 1987 ARA criteria; 78% had active disease, and the median duration of arthritis was 13 years. The aim of the present study was to determine whether anti-CB10 was present in the serum of patients with early RA, and to compare the frequency of anti-CB10, anti-CCP and RF, using stored sera from a community-based prospective study of early inflammatory arthritis from first referral to a specialist rheumatologist. Sera were tested from 82 patients for whom the final diagnosis was early RA to examine the associations of these three autoantibodies with the outcome: remission, persisting non-erosive arthritis, or development of erosions (Table 1). The study was carried out before the development of biologic therapies [52] and before anti-CCP was included among the American College of Reumatology (formerly ARA) criteria. 

## 2. Results

### 2.1. Frequency of Autoantibodies to CB10 in Established RA

Receiver operating characteristic (ROC) curve analysis was performed to establish a cut-off for the anti-CB10 ELISA, using sera from 52 patients from a cross-sectional study of long-standing erosive RA and 136 control sera. The area under the ROC curve was 0.738 (95% CI 0.669 to 0.799). Taking a level of specificity of 95%, a value greater than 48 units was considered positive; under these conditions, 34 (65%) of the RA sera were positive, and six of 136 controls (4%, 3/63 blood donors, 1/31 systemic lupus erythematosus (SLE), 2/42 other unspecified patients).

### 2.2. Autoantibodies to CB10 in Early RA

**Figure 1:**
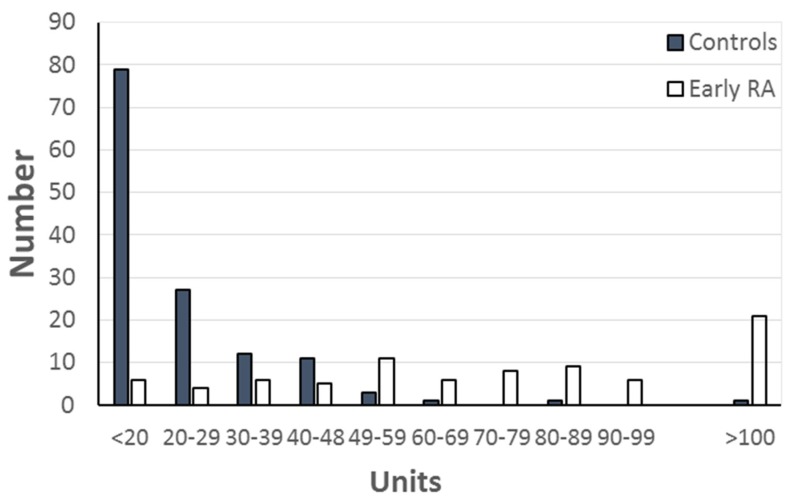
Distribution of levels of anti-CB10 in 82 patients with early RA and 136 controls. The upper limit of normal was 48 units, based on ROC plot analysis of the controls and 52 patients with long-standing RA. Anti-CB10 in the six positive controls were in two sera from patients with SLE (52 and 54 units), two biochemistry discards from patients with unspecified diagnoses (60 and 87 units), and two blood donors (54, 130 units).

**Figure 2:**
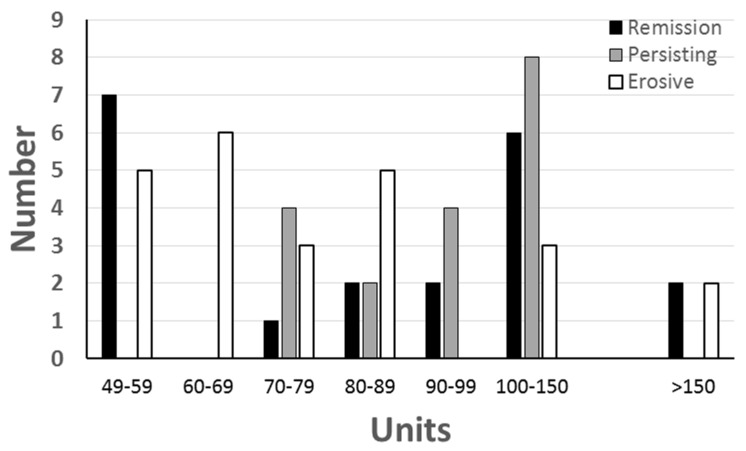
Distribution of levels of antibodies to CB10 at onset among 65 patients with early RA who had anti-CB10, grouped according to outcome—remission, persisting non-erosive arthritis or erosive arthritis.

Antibodies to CB10 were compared in the initial serum sample from 82 sera from patients with early arthritis, and the 136 control sera (Figure 1). Using the previously established value of 48 units to define the upper limit of normal, 62 (76%) of the patients were positive compared with 65% of the patients with long-standing RA. The frequency of anti-CB10 at presentation was not related to the progress of the arthritis, and most patients had anti-CB10, although the frequency was highest in those who attained remission (87% remission; 67% persisting; 75% erosive) (Figure 2, Table 2). There was no association between the presence of anti-CB10, and either one or two copies of the SE. 

### 2.3. Anti-CCP in Early Inflammatory Arthritis

For anti-CCP, 44 (54%) of the patients with early arthritis had antibodies, compared with 36 (69%) of the patients with long-standing RA. The frequency of anti-CCP at disease onset was increased among those patients who developed erosive arthritis when compared with those with persisting non-erosive arthritis and was lowest in those who attained remission (remission 22%, persisting non-erosive 52%, erosive 78%), but the levels of anti-CCP among positive sera were similar in each group. Thus, the median level and range of positive anti-CCP among five patients who remitted was 835 (300–2000), compared with 516 (60–3100) for 14 patients with persisting arthritis and 814 (35–3420) for 25 patients with erosive arthritis. Levels of anti-CCP were not related to length of time before the development of erosions. 

Among the total group of 69 patients for whom HLA typing was available, there was a significant association between the SE, and anti-CCP, and 26 of 40 patients (65%) with the SE had anti-CCP, compared with 10 of 29 (34%) without (χ^2^ = 6.27, *p* = 0.0122), consistent with the reported linkage between the SE and enhanced antigen presentation of citrullinated peptides [31]. However, the 10 patients with anti-CCP but without the SE were all DR2 (*n* = 7) and /or DR7 (*n* = 4), and seven of these had erosive arthritis. High levels of anti-CCP have been associated with HLA DRB1*15 (previously recognised with HLA DRB1*16 as DR2) in patients with RA who lack the SE [53]. 

### 2.4. Rheumatoid Factor in Early Inflammatory Arthritis

RF was present in the initial serum from 47 (57%) of the patients with early arthritis, compared with 67% of patients with long-standing arthritis. Like anti-CCP, RF occurred most frequently in erosive arthritis, and least in those patients who attained remission (remitting 30%, persisting non-erosive 67%; erosive 69%).

### 2.5. Predictive Value of Autoantibodies

**Figure 3:**
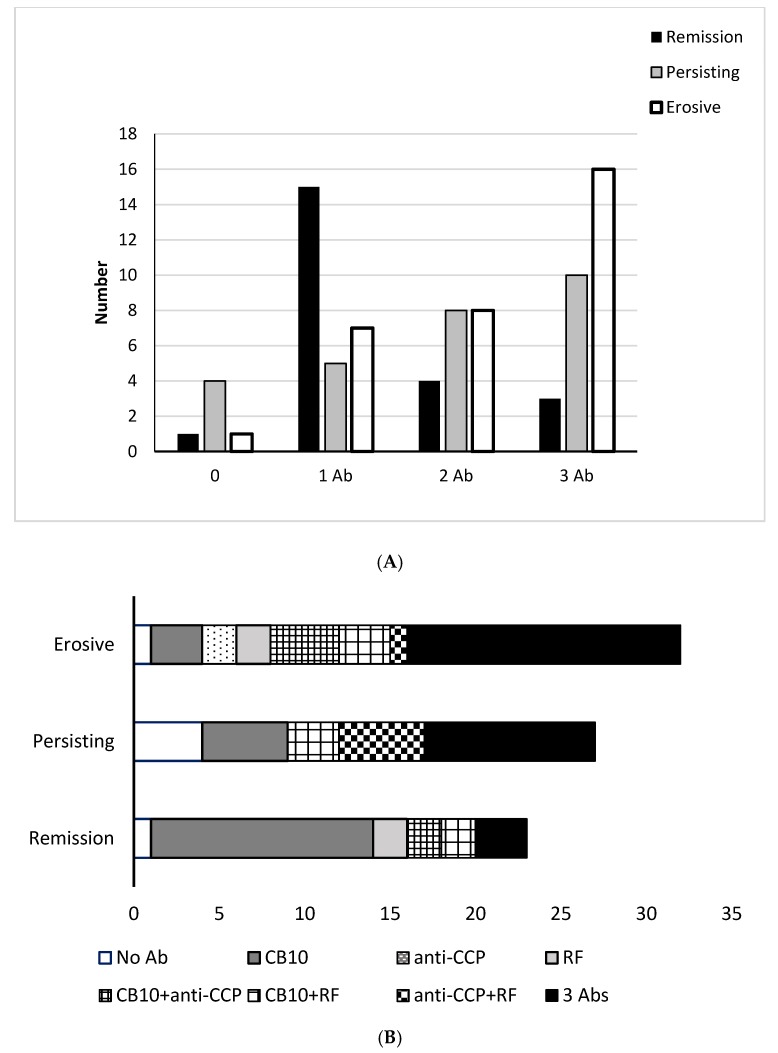
(**A**) Outcome in the 82 patients with early RA according to the number of autoantibodies detected initially; and (**B**) combinations of autoantibodies in the patients with early RA grouped according to outcomes.

Despite the increased frequency of both anti-CCP2 and RF with more severe disease, none of the autoantibodies alone was sufficient to predict the course of the disease. Combinations of antibodies at presentation were more informative, and generally, the more autoantibodies that were detected, the poorer the outcome for the patient (Figure 3A). Six patients had no detectable antibodies, and 27 had a single antibody, of whom 21 had anti-CB10, two, both with erosive arthritis, had anti-CCP and 4 patients had RF (Figure 3B). Of the 33 patients who had no autoantibodies or only one of the three, 16 (48%) attained remission, and eight (24%) developed erosive arthritis, whereas of 29 patients who had three autoantibodies, three (10%) attained remission, and 16 (55%) developed erosive arthritis. 

### 2.6. Sequential Changes in Autoantibodies

Levels of autoantibodies were measured in sequential sera from 20 patients, of whom five attained remission, six had persisting non-erosive arthritis, and nine had erosive arthritis. Although the levels of anti-CB10 fluctuated markedly in some patients but remained steady in others, overall there was a trend to lower levels of anti-CB10 as the disease progressed, and whereas 18 of the 20 (90%) patients had antibodies initially, only 11 (55%) had antibodies in the final sample (χ^2^ = 4.20, *p* = 0.04). Complete loss of anti-CB10 was particularly associated with progression to erosive arthritis, and of the nine patients with erosive arthritis who were examined sequentially, six became negative for anti-CB10 at the end of the study, and two patients were negative throughout.

In contrast to the decrease in anti-CB10 reactivity seen in the patients followed sequentially, levels of anti-CCP among the 10 patients who had anti-CCP initially (four persisting non-erosive, six erosive), remained stable whether low level (<100), mid-level (>100 < 1000), or high level (>1000), and none lost reactivity. Four patients developed anti-CCP during the follow-up. In each case the patients had anti-CB10 initially. One patient who developed erosive arthritis had anti-CCP (800 units) when tested at one year. Both tests were still positive at four years, although the levels of anti-CB10 were falling, and both tests were negative by eight years. A second patient with persisting arthritis had a single high anti-CCP (550 units) at five years; the test was again negative at six years. Two further patients, one of whom remitted and one who developed erosive arthritis, had transient low levels of anti-CCP (<70 units), although both were strongly positive for anti-CB10.

Among the 20 patients for whom sequential sera were tested, 11 patients had RF initially (five persisting non-erosive, six erosive). There was no consistent pattern of changes observed in these patients. Three of these patients recorded at least one negative test, and the test became positive in one patient.

## 3. Discussion

RA is a disease of variable onset and expression that reflects its multigenic origin [54]. It has been considered to be an immune complex disease, based on the local production of autoantibodies, historically rheumatoid factor, with immune complexes and a low level of complement within the joint [6,11]. For many years type II collagen has been proposed as the elusive joint-specific autoantigen [55], but studies have been limited by the lack of reproducible and reliable assays for antibodies to CII. Using an assay to detect antibodies to the CB10 peptide of CII, we reported antibodies in the sera of 88% of patients with long-standing severe RA [51]. In the present study, we have examined the potential role of autoantibodies in the development of RA and the clinical course in 82 subjects with early inflammatory arthritis, most fulfilling the 1987 criteria [1] and excluding other inflammatory arthritis, prior to the availability of biologic therapies. These subjects were followed prospectively over a period of up to 10 years, employing assays that were comparable in sensitivity and specificity in established RA, to compare the presence of three autoantibody populations, anti-CB10, anti-CCP and RF in stored sera taken at the onset of symptoms. The outcome was categorised as attainment of remission, persistence of arthritis, or development of erosions. No similar study in early RA has been reported, and although the number of subjects studied was small, current emphasis on the importance of early treatment and the advent of new biologics would now make such a study difficult to perform. 

Of the three autoantibodies, anti-CB10, used as a measure of anti-CII, was detected in 76% of patients overall, including 87% of 23 patients who attained remission, 12 of whom had only anti-CB10, and was the most abundant of any of the three autoantibodies. Levels of anti-CB10 were higher in the patients with early RA than in patients with established disease and in the 20 patients for whom sequential samples were available levels decreased with time. Similar falls in levels of circulating anti-CII have been observed by others [33,34,35,56] and a possible explanation for this is that serum antibodies to CII are progressively removed from the circulation and sequestered within the cartilage as damage to the matrix and proteoglycan loss exposes more CII to complex with the antibody. Elution of anti-CII from cartilage of subjects with RA [57] lends support to this notion. These results are consistent with a role for CII as the elusive joint-specific autoantigen in RA, but the frequency of anti-CII in patients who remitted suggests that this antibody alone is insufficient to promote severe arthritis.

In contrast to anti-CB10, anti-CCP occurred in only 54% of the patients overall but was strongly associated with the development of erosions, being present in 78% of those who developed erosive arthritis. Anti-CCP production was linked to the SE, but not all patients who developed erosions had the SE and the development of erosions were more strongly associated with anti-CCP than with the SE, possibly as a result of an additional HLA-association with DR15 [53]. Anti-CCP represents reactivity with a wide range of citrullinated antigens, few of which may be expressed in the joint, and in contrast to the changes in levels of anti-CB10, levels of anti-CCP in serum showed very little change.

The third autoantibody, RF, was present in 57% of patients at onset, and in 69% of those who developed erosive arthritis. Unlike anti-CCP, serum levels of RF varied inconsistently amongst those who were followed sequentially. Possibly, it plays a role in immunoregulation by cross-linking Fc-regions of antibodies, increasing the net avidity and specificity of antibodies of low affinity, forming immune complexes and activating complement. Accordingly, it could play an essential role in removing pathogens [13]. RF has been particularly associated with antibody responses to repetitive, highly-ordered patterns of epitopes, such as those that occur in collagen [58].

Taken together, all of these autoantibodies could contribute to immune complex formation. Once on-going inflammation is established, the specificity of the initiating autoantibodies (anti-CII) may become an ongoing but minor component of the flagrant inflammatory response as RF and antibodies to various citrullinated antigens become the driving force for the feedback loop involving inflammation, cytokine production, matrix damage, and chondrocyte activation. In the present study, the presence of all three autoantibodies, anti-CB10, anti-CCP, and RF, was a predictor of outcome: 13% in those who attained remission, 37% in those with persistent disease, and 50% in those with erosions. For each of these antibody systems, the autoantibodies would contribute directly to immune complex mediated damage according to their class and subclass by cellular interactions involving binding to Fc-receptors, and complement activation. 

This view of RA as an immune complex-mediated disease of articular joints has been supported by the development of models of antibody induced arthritis, both CAIA, induced using mAb to CII [48], and also K/BxN serum-transfer arthritis (K/BxN STA) [59], induced using mAb derived from mice of the K/BxN transgenic strain that spontaneously develop severe arthritis. In the K/BxN STA model autoantibodies are to glucose-6-phosphate isomerase, a ubiquitously expressed intracellular enzyme that is expressed on the cartilage surface in mice. In both of these models, despite the substantial differences in the inductive phase of the arthritis there are common pathways of expression and similar outcomes in which the inflammatory response is driven by autoantibodies that form immune complexes within the joint, activation of innate immune cells, such as neutrophils and macrophages, and immune mediators, including cytokines, chemokines, complement, Toll-like receptors, Fc receptors, and integrins. In both models, the arthritis exhibits leukocyte invasion, synovitis, pannus formation, and cartilage and bone destruction similar to that seen in RA [48,55,59]. These symptoms are entirely antibody-initiated, representing the effector arm of an immune response primarily driven by Fc-mediated interactions.

The repetitive sequence of epitopes on the CII molecule of the cartilage favours formation of multimeric immune complexes and activation of the complement system. This attracts a cellular infiltrate resulting in the release of cytokines, chemokines, and various enzymes which contribute to inflammation and further damage to cartilage and surrounding tissue. Particularly relevant is the incursion of polymorphonuclear leucocytes, monocytes, and macrophages that generate calcium-dependent peptidylarginine deiminase enzymes [16,60] with the capacity to citrullinate arginine molecules on the autoantigenic motifs of CII [49], as well as a range of peptides that are part of the joint structure, but are not specific to joints. These citrullinated neoepitopes result in T cell responses under the influence of the SE [31], stimulating localised ACPA production within the joint [61] and the development of antibodies that are primarily determined by the presence of the SE rather than the disease itself [62]. However, a response to a neoepitope seen as a “foreign” antigen may well be poorly regulated, with enhanced potential to drive local immune complex formation. It then becomes a marker of severe disease [63] as confirmed by our finding of 78% of subjects who developed erosions.

In both CAIA and K/BxN STA, combinations of mAb reactive with different epitopes on the antigen more readily induce arthritis than any single mAb alone [64,65] and this may merely represent enhanced ability to form mAb/Ag multimers, with increased FcR and complement activation. However, although these diseases can be considered as induced by immune complexes, in which Fab-specificity of the antibody is secondary to immune activation by Fc-mediated effects, further levels of complexity are contributed by the specific Fab interactions of epitopes recognised and this complexity may be what is seen in the polyclonal response in RA. In CAIA, both arthritogenic and protective mAbs have been identified, and epitope analysis has identified regions of the CII molecule where mAb binding causes direct cartilage damage [44,46,66,67,68,69,70]. 

Articular cartilage has an unusual structure, as it is a relatively acellular tissue in which a small number of chondrocytes are surrounded by an abundant extracellular matrix. It comprises a network of fibrils of CII and other less abundant collagens, in which are entrapped negatively-charged glycosaminoglycans and proteoglycans, particularly hyaluronan and aggrecan, and other minor, but important, components that interact to maintain the integrity of the matrix and allow frictionless articular movement. The chondrocytes are entirely responsible for the formation and synthesis of the matrix. In cartilage explant cultures, in the absence of inflammatory mediators, several arthritogenic mAbs to CII cause marked proteoglycan loss from the cartilage surface where the antibody penetrates, with progressive denaturation and damage to the collagen matrix [46,69,71].The same arthritogenic mAbs also affect synthesis of new matrix by chondrocytes in vitro, with effects both on cell morphology and on collagen synthesis that differ according to the specificity of the mAb [46,70]. These changes are Fab-mediated, do not require cells other than chondrocytes, and are much more striking in cartilage where the chondrocytes have been killed by freeze-thawing [68]. It is likely that mAb derived from mice with CIA interfere with interactions within the cartilage, either between collagen and chondrocytes, or collagen and other matrix components. In adult cartilage, chondrocytes are relatively inert, and matrix turnover occurs only slowly, but the balance between damage and repair in culture is suggestive of the relapsing-remitting progress that is often seen in human RA. Moreover, as chondrocyte activity and matrix synthesis decreases with age, an imbalance between damage and repair as a result of antibody binding is consistent with the increased incidence of RA with age. Similar studies have not been performed with ACPA or RF, but the arthritogenic anti-CII mAb react with surface-exposed epitopes on CII that contain arginine, and equivalent ACPA mAb reactive with the citrullinated epitopes have been derived that enhance the development of arthritis [50,72], and may well have similar disruptive structural effects. 

Although this is the only study we are aware of that has examined the three autoantibody populations in early RA a major limitation of this study, and of others, is that we have measured antibodies in serum, not synovial fluid. It would be both impractical and unethical to attempt to replicate such a prospective study using synovial fluid, but we are mindful that the site of inflammation is within the joint. Synovial fluid and serum represent two different compartments, with limited exchange between them and levels of IgG, IgM, or IgA in normal synovial fluids are a fraction of the levels in serum [73,74]. As each of the autoantibodies we have studied has been shown to be produced locally within the joint and is more abundant at the site of inflammation and damage, measurements of serum autoantibodies may be misleading and represent only a ‘spill-over’ from the site of the lesion in the joint. Despite this reservation, the measurement of the triad of autoantibodies—anti-CB10, anti-CCP and RF—has provided insight into the outcome of RA in subjects diagnosed with early inflammatory arthritis, and in nominating CII as the putative autoantigen implicated in the failure of immunological tolerance in RA, we have outlined the key steps in the evolution of the efferent arm of the autoantibody response. 

Finally, an important aspect of this study is the observation that anti-CB10 were present very early in the course of disease in these patients. The diagnosis of early inflammatory arthritis as RA was a clinical diagnosis made in the course of treatment by a specialist rheumatologist (AS) after exclusion of other potential diagnoses. Not all patients fulfilled the ARA criteria for RA when first seen, noting that nodules and erosions are late sequelae, and four criteria require symptoms to be present for six weeks, whereas some patients in each group were first seen and commenced disease-modifying drugs within that period. We have rarely detected anti-CB10 in other diseases associated with arthritis, including osteoarthritis and psoriatic arthritis [51], or in the present study, in patients with SLE. The increasing emphasis of the importance of early treatment for RA, and the recognition that ARA criteria are not helpful for diagnosis in the first 12 months of the disease [75] suggest that the detection of autoantibodies to CII has important implications, clinically. The presence of these autoantibodies in early arthritis points to an autoimmune reaction affecting articular joints and should alert the clinician to the likelihood of a developing rheumatoid arthritis. Tests, including those determining the profile of autoantibodies, can then be undertaken to establish diagnosis, and implement treatment appropriate to the findings and the follow-up required.

## 4. Materials and Methods

### 4.1. Patients

Serum samples were tested from 82 patients diagnosed with early RA who were being followed as part of a prospective study of recent-onset inflammatory arthritis [52]. The patients were from the rheumatology community-based private practice of one of the authors (AS) who performed all assessments. The diagnosis was based on the clinical assessment of the rheumatologist and patients with evidence of other recognized inflammatory arthritis, such as psoriatic arthritis, crystal arthritis, or other connective tissue diseases, were excluded. Inclusion diagnosis were two or more swollen and painful joints present for at least two weeks. Patients were not required to fulfil 1987 ARA criteria [1]. Median duration of arthritis at baseline was three months (range: 1 week–8 months) [51]. As part of this study, clinical assessments included the Ritchie articular index for tender joints [76], the total number of swollen joints, 1987 ARA criteria fulfilled, ESR, and presence of erosions in radiographs of the hands and feet assessed based on a Larsen score of 2 or more [77](Table 1). Positivity for the DRB1 shared epitope (SE) that has been linked to the pathogenesis of RA was assessed by DRB1 genotyping performed as a two-step process using PCR amplification followed by hybridization with sequence-specific oligonucleotides [52]. The initial serum samples tested were obtained within eight months of the onset of symptoms (median: 3 months; range: 2 weeks–8 months). All patients were followed prospectively for at least two years (median: 4.5 years; range: 2–10 years); 32 (40%) developed erosive arthritis, usually within three years, and 21 (26%), including two with erosions, attained remission that was defined as no evidence of clinical synovitis and no requirement for disease-modifying drugs or corticosteroids for 12 months [78]. Details of the groups of patients are shown in Table 1. To examine changes in antibody levels, 123 additional sera were tested from 20 of the patients with early RA, with a median follow-up of five years (range: 2–8 years). 

For comparison, sera from 52 patients with long-standing RA, according to the 1987 criteria of the American College of Rheumatology (ACR) were included to determine the frequency of autoantibodies in established disease. These patients had a median age of onset of 45.5 years (range: 25–74 years), and duration of 15 years (range: 2–61 years). They were included as part of a cross-sectional study of rheumatoid arthritis for whom clinical and genetic data have previously been reported [79,80]. All had erosions at the time of testing. One hundred thirty-six additional controls included 63 sera from blood donors attending the Red Cross Blood Bank in Melbourne, and 73 sera obtained under code from pathology laboratories. Of these, 31 were from patients with systemic lupus erythematosus, all of whom had antibodies to ds-DNA, and 42 were a random selection of sera discarded by the hospital biochemistry laboratory and, for these sera, no clinical details were available. Sera from the patients with RA were collected over a 10-year period, and stored at −20 °C for later testing. All tests were performed at the same time, and sequential sera from a single patient were tested in the same assay. 

All subjects gave their informed consent for inclusion before they participated in the study. The study was conducted in accordance with the Declaration of Helsinki, and was approved by the Royal Melbourne Hospital Board of Medical Research and Ethics Committee of the Royal Melbourne Hospital. The Monash University Human Research Ethics Committee (MUHREC) approved the use of the stored sera under the provisions of “Ethics Approval for the Use of Stored Human Tissue Samples”.

### 4.2. Preparation of CB10

Bovine type II collagen was prepared from cartilage of the nasal septum by pepsin digestion and differential salt precipitation as described previously [81], and digested with cyanogen bromide [82]. CB10 was purified using cation exchange chromatography at 42 °C on carboxymethyl cellulose (CM-cellulose, Whatman CM-62) equilibrated in 0.02 M sodium citrate, and elution with a 0.01 M–0.16 M linear salt gradient. To further purify CB10 from CB11, CB8 and CB9,7 fractions were pooled, desalted and lyophilised, and applied to a P-30 column (100–200 mesh, exclusion limit 40 kD, globular proteins. Biorad, Hercules, CA, USA) equilibrated in 0.1 M acetic acid. To determine the purity of the peptides, fractions were run on 15% tricine gels and stained with 0.2% (*w*/*v*) Coomassie blue. Purified CB10 was renatured by stepwise cooling from 20 °C to 0.2 °C [83]. 

### 4.3. Detection of Antibodies to CB10 by ELISA

Antibodies to CB10 were measured using a previously developed ELISA in which intact CII was replaced with the CB10 peptide. Microtiter plates were coated overnight at 4 °C with 100 μL of 20 μg/mL CB10 in PBS pH 7.4, and blocked for 2 h with 200 μL of 1% skimmed milk in PBS (SMPBS) containing 0.05% Tween 20. Sera were tested in duplicate at a dilution of 1:200 in the blocking buffer, using a 2 h incubation. Antibodies were detected using horseradish peroxidase-conjugated (HRP) sheep anti-human IgG (Amrad, Hawthorn, Australia) diluted 1:2000 in SMPBS without Tween 20. As CB10 is unstable above 20 °C, all incubations to this point were performed at 4 °C, using cold buffers. Plates were equilibrated to room temperature for 10 min, and colour development was carried out at room temperature using 100 μL 3,3′, 5,5′ tetramethylbenzidine liquid substrate for ELISA (Sigma-Aldrich, Sydney, Australia); absorbance was read at 655 nm at exactly 10 min. The monoclonal antibody M2139 that reacts with the J1 conformational epitope in CB10 [44], was included to confirm that the CB10 retained its correct conformation. It was defined to contain 100 units of reactivity, and used as a standard with titration from 1:200 on every plate.

### 4.4. Measurement of Autoantibodies to Citrullinated Protein Antigens

ACPA were measured using the commercial anti-CCP2 assay from Euro-Diagnostica that is based on a panel of cyclic citrullinated peptides, according to the manufacturer’s instructions, with a manufacturer-derived cut-off for positivity of 25. 

### 4.5. Measurement of RF

The presence and titer of rheumatoid factor was measured by external laboratories as part of the initial clinical assessment of the patients, but stored sera were retested for this study using a Roche turbidimetric assay for RF according to the manufacturer’s instructions, with a manufacturer-derived cut-off for positivity of 14. 

### 4.6. Statistical Analysis

MedCalc statistical software [84] was used for derivation and analysis of receiver operating characteristic curves (ROC curves) for the autoantibodies. 

## 5. Conclusions

RA is a common chronic inflammatory disease of articular joints that often leads to joint destruction. The cause is unknown, but is best explained by autoimmunity, mainly based on the presence of autoantibodies and compelling evidence from experimental animal models. However, in autoimmunity the induction and effector mechanisms presently lack transparency and, in RA, the autoantigen implicated in the failure of immunological tolerance is still in dispute. In the present study we propose the arthritogenic response commences with an *autoimmune response* to CII and this results in immune complex formation due to autoantibody binding to repeated epitopes on the large triple helical molecule of CII on the articular joint surface. This destabilizes cartilage, attracts cells associated with inflammation, and results in an independent *neoimmune response* to citrullinated proteins that include not only CII, but a number of ubiquitous proteins that are under the influence of the SE. Formation of immune complexes result in the development of RF, which may either potentiate the immune response or have a role in clearance. This sequence of events is supported by our finding of a high frequency of anti-CB10 representative of anti-CII in subjects with early inflammatory arthritis, the persistence of anti-CCP as severity increases, and the presence of “the full-house” of autoantibodies—anti-CB10, anti-CCP, and RF—in the subjects with severe disease.

## Figures and Tables

**Table 1:** Patients with arthritis grouped according to outcome. Results are shown as median (range).

	Early inflammatory Arthritis *			Longstanding Erosive RA
	Remission	Persisting Non-Erosive	Erosive	
Number	23	27	32	52
F:M (%F)	13:10 (57%)	14:13 (52%)	25:7 (78%)	36:16 (69%)
Age at onset (years)	71 (40–81)	56 (25–81)	58.5 (20–79)	45.5 (25–74)
Duration before presentation (months)	3 (1–7)	3 (0.5–7)	3 (0.3–8)	-
Duration before remission (months)	12 (4–36)	-	-	-
Duration of follow-up (years)	4 (2–7)	4 (2–10)	5 (2–10)	15 (2–61)
Joints swollen at presentation	7.5 (0–17)	4 (1–22)	9 (1–47)	
Joints swollen 24 months	0 (0–2)	0 (0–14)	3 (0–17)	
Ritchie at presentation	4.5 (2–9)	4 (0–18)	6 (2–21)	
Ritchie 24 months	0 (0–4)	2 (0–16)	3 (0–22)	
ESR^**^ at presentation	39 (12–94)	28 (1–82)	38 (8–111)	
ESR 24 months	7.5 (1–112)	14 (1–106)	25 (1–76)	
ARA criteria at presentation	3 (1–5)	4 (2–6)	4 (1–7)	
ARA criteria 24 months	0.4 ± 0.8 (0–3)	1 (0–5)	3 (0–6)	
Shared epitope (%)	11/21 (52%) 2NT	11/20 (55%) 7NT	18/28(64%) 4NT	

NT not tested; * patients with evidence of other recognized inflammatory arthritis such as psoriatic arthritis, crystal arthritis or other connective tissue diseases have been excluded; **Erythrocyte sedimentation rate

**Table 2:** Autoantibodies in patients with arthritis and controls.

	Early Inflammatory Arthritis			Longstanding RA	Controls
	Remission	Persisting Non-Erosive	Erosive		
Number	23	27	32	52	136
Anti-CB10	20 (87%)	18 (67%)	24 (75%)	34 (65%)	6 (4%)
Anti-CCP	5 (22%)	14 (52%)	25 (78%)	36 (69%)	0 (0%)
Rheumatoid factor	7 (30%)	18 (67%)	22 (69%)	35 (67%)	12(9%)
Number of antibodies (3:2:1:0)	3:4:15:1	10:8:5:4	16:8:7:1	22:23:5:2	0:0:18:118
